# Synthetic self-assembling ADDomer platform for highly efficient vaccination by genetically encoded multiepitope display

**DOI:** 10.1126/sciadv.aaw2853

**Published:** 2019-09-25

**Authors:** Charles Vragniau, Joshua C. Bufton, Frédéric Garzoni, Emilie Stermann, Fruzsina Rabi, Céline Terrat, Mélanie Guidetti, Véronique Josserand, Matt Williams, Christopher J. Woods, Gerardo Viedma, Phil Bates, Bernard Verrier, Laurence Chaperot, Christiane Schaffitzel, Imre Berger, Pascal Fender

**Affiliations:** 1 Institut de Biologie Structurale (IBS), Université Grenoble Alpes, CNRS, CEA, 71 Avenue des Martyrs, 38042 Grenoble, France.; 2 Bristol Research Centre for Synthetic Biology BrisSynBio, School of Biochemistry, University of Bristol, 1 Tankard’s Close, Bristol BS8 1TD, UK.; 3 Imophoron Ltd., Unit DX, St. Philips Central, Albert Road, Bristol BS2 OXJ, UK.; 4 Laboratoire de Biologie Tissulaire et d'Ingénierie Thérapeutique (LBTI), UMR 5305, Université Lyon 1, CNRS, Institut de Biologie et Chimie des Protéines (IBCP), Lyon, France.; 5 Cancer Target and Experimental Therapeutics, Institute for Advanced Biosciences, INSERM U1209, CNRS UMR5309, Université Grenoble Alpes, 38700 Grenoble, France.; 6 Advanced Computing Research Centre, University of Bristol, 31 Great George Street, Bristol BS1 5QD, UK.; 7 Oracle Cloud Development Centre, Tower Wharf, Cheese Lane, Bristol BS2 2JJ, UK.; 8 Immunobiology and Immunotherapy in Chronic Diseases, Institute for Advanced Biosciences, INSERM U1209, CNRS UMR5309, Université Grenoble Alpes, Etablissement Français du Sang-Rhone-Alpes, 38700 Grenoble, France.; 9 Max Planck-Bristol Centre for Minimal Biology, Cantock’s Close, Bristol BS8 1TS, UK.

## Abstract

Self-assembling virus-like particles represent highly attractive tools for developing next-generation vaccines and protein therapeutics. We created ADDomer, an adenovirus-derived multimeric protein-based self-assembling nanoparticle scaffold engineered to facilitate plug-and-play display of multiple immunogenic epitopes from pathogens. We used cryo–electron microscopy at near-atomic resolution and implemented novel, cost-effective, high-performance cloud computing to reveal architectural features in unprecedented detail. We analyzed ADDomer interaction with components of the immune system and developed a promising first-in-kind ADDomer-based vaccine candidate to combat emerging Chikungunya infectious disease, exemplifying the potential of our approach.

## Teaser

ADDomer is a synthetic, self-assembling, virus-like particle platform that enables highly efficient vaccination.

## INTRODUCTION

Self-assembling protein-based nanoparticles are highly attractive tools for a broad range of biomedical applications, including vaccine development and cancer therapy (*1*–*4*). Present in all kingdoms of life, they form supramolecular architectures with unique properties (*4*), including spontaneous self-organization from simple precursor protomers amenable to engineering. Moreover, the particle size is generally in the range of pathogens, notably viruses, which the immune system has evolved to strongly react against (*5*, *6*). Protein-based nanoparticles often adopt quasi-spherical shapes encapsulating a central cavity that can carry cargo, rendering them suitable to deliver drugs (*3*). Virus-like particles (VLPs) are made of many copies of identical building blocks resulting in highly repetitive surfaces, providing opportunities to display pathogen-derived epitopes. These are often oligopeptide sequences, on their own generally not immunogenic enough to elicit a strong immune response resulting in protection (*5*, *6*). However, if coupled to self-assembling protomers forming the VLP, then peptide epitopes can reach very high densities on the VLP, potentially able to trigger B cell receptor clustering and cross-presentation for facilitation of a strong immune response (*5*, *7*). Notably, VLPs can act as self-adjuvants, alleviating the need to supplement vaccine formulations with additional adjuvanting reagents that can have undesired side effects (*8*).

VLPs are intensely researched toward new and better vaccines and therapeutics against acute and chronic diseases (*2*, *3*, *9*–*11*). An ideal VLP-based vaccine would be safe, free of nucleic acid contaminants, amenable to engineering, well produced recombinantly, easy to purify, available in large amounts, self-adjuvanting, and capable of eliciting a strong immune response to pathogen-derived antigens presented in a native conformation at high density on the particle surface. A major aspect is thermotolerance—many vaccines today require refrigeration, rendering storage and deployment dependent on a functioning cold chain, posing problems in remote or less affluent regions (*12*). Thus, thermostability would be a highly advantageous asset for any VLP vaccine.

Human adenoviruses are among the most widely used vectors in gene therapy (*13*). The icosahedral adenovirus capsid comprises distinct proteins arranged in hexons, pentons, and fiber extrusions. The penton-forming protomer of certain adenovirus serotypes can self-assemble into a symmetric, hollow dodecahedron adopted by 12 pentons comprising altogether 60 protomers (*14*), effectively representing a proteinaceous, nonenveloped VLP. We showed previously that human adenovirus serotype 3 (Ad3) dodecahedron can be produced in baculovirus-infected insect cells (*15*) and can retain the adenovirus-like ability to penetrate epithelial cells (*16*). We studied the structure and mechanism of the Ad3-derived dodecahedron, investigating its potential as a delivery system for DNA, proteins, and chemical compounds (*17*–*19*). Encouraged by these promising proof-of-concept results, we set out here to create a synthetic, engineered Ad3-derived stable dodecahedron-forming protomer for highly efficient multiepitope display and delivery toward a next-generation ADDomer VLP platform for vaccines and protein therapeutics.

## RESULTS

### ADDomer multiepitope display and delivery platform

**Fig. 1:**
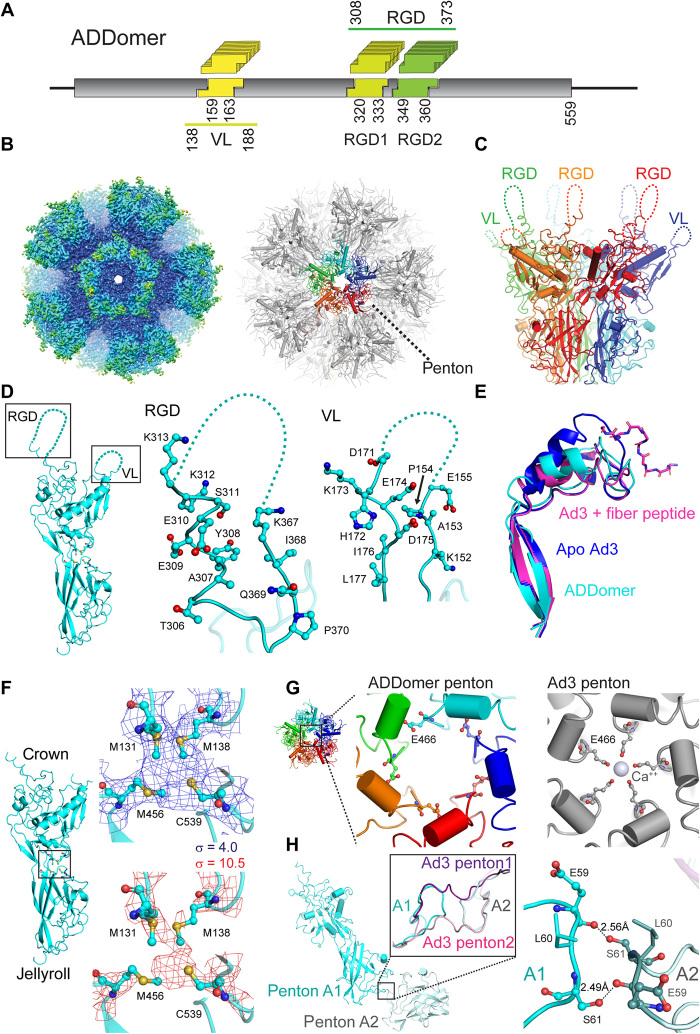
Cryo-EM structure of ADDomer. (**A**) ADDomer design. Three evolutionary nonconserved segments (boxed) in the protomer were engineered by BioBrick design into exchangeable cassettes. One cassette (yellow) is located within the VL, while two cassettes, RGD1 (light green) and RGD2 (dark green), are located within the loop containing a functional RGD tripeptide sequence. Numbers indicated amino acid boundaries of BioBrick cassettes as well as VL and RGD loop. (**B**) Left: A 3.5-Å cryo-EM map of the ADDomer particle formed by 60 protomers. The rigid core is colored blue, and more flexible regions comprising the loops are colored cyan and green. Right: The corresponding atomic model is shown in gray. One penton (center) is highlighted, with individual protomers colored red, orange, green, cyan, and blue, respectively. (**C**) Side view of a penton formed by five protomers. Flexible VL and RGD loop are drawn in dashed lines. (**D**) Closeup view of the RGD loop and VL in ADDomer. Residues 156 to 170 in VL and 314 to 366 in RGD loop in the BioBrick format maintain their flexibility, essential for functionalization by epitope insertion. (**E**) Superimposition of the fiber-binding region in the ADDomer cryo-EM structure (cyan) with apo Ad3 [Protein Data Bank (PDB) ID: 4AQQ; marine blue] and fiber peptide–bound Ad3 (PDB ID: 4AR2; magenta) crystal coordinates. The fiber peptide (PDB ID: 4AR2) is drawn in a ball-and-stick representation. (**F**) Individual ADDomer protomer is shown in a side view (left) with a putative metal-binding cluster boxed in between the crown (top) and jellyroll fold (bottom) domains. Zoomed-in views of the boxed region (right) depict the atomic model in the EM density contoured at two different levels, σ = 4 (blue) and σ = 10.5 (red). Four juxtaposed sulfurs (yellow spheres) likely coordinate a metal ion. (**G**) Left: Central channel of an ADDomer penton. Right: Ca^2+^ coordination as seen previously in the Ad3 penton base protein crystal structure (PDB ID: 4AR2) is not observed. The helices fencing the central channel are rearranged, resulting in a different conformation of E466. (**H**) Interface between two pentons (A1 and A2) in the ADDomer (cyan). The zoomed-in view depicts the boxed region with the corresponding Ad3 crystal structure (magenta) superimposed. Domain swapping is not observed in the ADDomer. Instead, this interface is stabilized by mutual hydrogen bonds between S61 side chains and E59 peptide backbones from neighboring protomers (right).

Comparison of primary sequences of penton-forming protomers from many adenovirus serotypes revealed particularly two regions of high variability in length and sequence, called variable loop (VL) and arginine-glycine-aspartic acid (RGD) loop. For instance, VL comprises 20 amino acids in Ad3 while only 9 amino acids in Ad41. RGD loop plasticity is even more pronounced in Ad12, spanning 11 residues, in contrast to 74 residues in Ad2. The VL has been shown previously to accommodate an influenza-derived immunogenic eptitope (*20*). The RGD loop contains a conserved tripeptide motif (-RGD-) mediating integrin-based internalization into target cells (*21*). We reasoned that these regions, VL and RGD loop, given their polymorphism, would be ideally suited to accommodate one or several antigenic epitopes each. Therefore, we redesigned the protomer-encoding gene adopting a “BioBrick” format (*22*) to facilitate multiple epitope insertions. We inspected the crystal structure of Ad3 dodecahedron (*17*) and identified suitable loci adjacent to secondary structure motifs flanking the loops. The RGD loop in wild-type Ad3 is considerably more extended than the VL. As the tripeptide is important for cell internalization, we decided to split the RGD loop into two sections before and after the RGD motif, which we kept unaltered. We inserted unique restriction sites to create three independent loci for epitope display, one in the VL and two in the RGD loop to facilitate insertion of synthetic DNAs encoding for antigenic epitopes of choice in the resulting plug-and-play multiepitope display platform, the ADDomer (Fig. 1A and figs. S1 and S2). We expressed ADDomer using MultiBac, the baculovirus/insect cell system we developed for complex protein biologics (*23*), resulting in highly purified ADDomer (fig. S1) with excellent culture yields (50 mg from a 50-ml culture) and negligible nucleic acid or endotoxin contamination.

### ADDomer structure and mechanism

Unlocking ADDomer to future structure-based design, we determined its molecular architecture by high-resolution cryo–electron microscopy (cryo-EM) (Fig. 1, B to H, and figs. S3 and S4). For parts of the process, we used public cloud resources, implementing image processing and refinement software for this purpose. Our cryo-EM structure, at up to 3.2-Å resolution, reveals ADDomer adopting the familiar dodecahedron, formed by 12 pentons in a quasi-spherical arrangement, with the RGD loop and VL densely decorating the particle surface (Fig. 1, B and C, fig. S3, and table S1). Our structure shows that the plasticity of the RGD loop and VL, essential for antigenic epitope presentation, is maintained in ADDomer (Fig. 1D).

Crystal structures of wild-type Ad3 dodecahedron with and without fiber peptide bound exist at lower resolution (3.8 and 4.8 Å, respectively) (*17*). Close inspection of our cryo-EM structure reveals notable differences. The fiber-binding cleft adopts a rearranged conformation, seemingly in between the geometries observed in the crystals of apo- and fiber-bound Ad3 dodecahedron (Fig. 1E) (*17*). The protomer represents a two-domain architecture, with a crown region comprising the VL and RGD loop abutting a jellyroll fold mediating multimerization (Fig. 1, D and F). In our cryo-EM structure, we observed additional density in between the crown and jellyroll domains, juxtaposed to four sulfur-containing amino acids (M131, M138, M458, and C539), consistent with a tetradentate coordination by a metal ion, possibly zinc or iron (Fig. 1F). We posit that a structural metal ion at this interdomain interface could be important for stabilizing this protein fold.

In the Ad3 dodecahedron crystal, a Ca^2+^ ion occupied a central cavity lined by glutamates (E466) in α-helices from different protomers (Fig. 1G). No corresponding density is observed by cryo-EM. The Ad3 crystallization conditions contained CaCl_2_, whereas we did not supplement Ca^2+^ during ADDomer purification to occupy this site. Consequently, glutamates E466 do not extend into the cavity, but the corresponding α-helices are slightly shifted in the ADDomer.

A hallmark in the Ad3 crystal structure was strand-swapping between extended N-terminal regions of neighboring protomers, deemed essential to dodecahedron structural integrity (*17*). Unexpectedly, we did not observe this strand-swapping by cryo-EM (Fig. 1H and fig. S4C). Rather, in ADDomer, two neighboring protomers engage in hydrogen bonds in the vicinity of the twofold axis, extending from the S61 side chain to the peptide backbone of E59 and vice versa. We can exclude that the differences observed are due to ADDomer amino acid additions and substitutions within the VL and RGD loop, as they are in the crown domain and thus sterically too distant to have a bearing on the N-terminal domain, or the formation of the hitherto unobserved metal binding site, respectively.

Strand-swapping and Ca^2+^ ions would conceivably stabilize the dodecahedron. We did not observe either of these features by cryo-EM and thus wondered about ramifications for ADDomer stability. Thermal shift assays evidenced a melting temperature of 54°C for ADDomer and no noticeable melting below 45°C (fig. S5A). We challenged ADDomer integrity by storing for weeks at room temperature, freezing and thawing, and incubating at 45°C. Negative-stain EM revealed uniformly stable ADDomer (fig. S5, B to E). Thus, we can conclude that ADDomer maintains highly advantageous thermotolerance, begging the question what exactly the source of this remarkable stability may be.

**Fig. 2:**
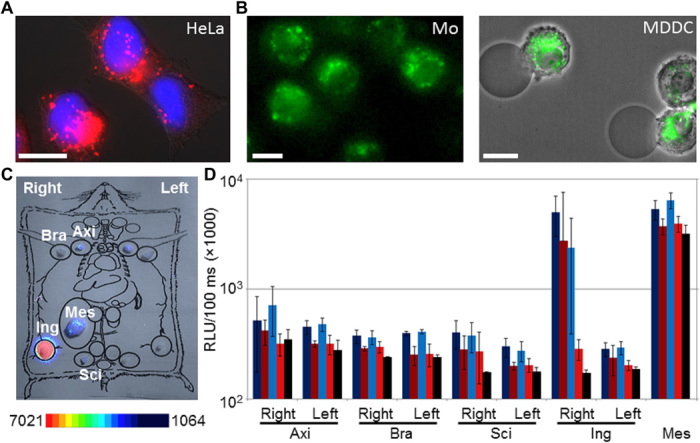
ADDomer internalizations. (**A**) HeLa cells incubated with ADDomer. 4′,6-diamidino-2-phenylindole–stained nuclei are colored blue. ADDomer detected by punctuate-specific immunofluorescence is colored red. Scale bar, 20 μm. (**B**) Human monocytes (Mo; left) and MDDCs (right) were incubated with Alexa Fluor 488–labeled ADDomer (green). Efficient uptake of ADDomer was observed by in all cell types tested. Scale bars, 20 μm. (**C**) Lymph node biodistribution of Alexa Fluor 680–labeled ADDomer in mice. Ex vivo imaging of the main lymph nodes from one representative mouse 5 hours after subcutaneous injection (10 μg) in the right leg is depicted in a schematic drawing (left). Specific fluorescence signal was quantified (for 100 ms), and intensities (in arbitrary units) are plotted in spectral colors (bottom). (**D**) Ex vivo quantification (for 100 ms) of ADDomer in isolated lymph nodes from a cohort of mice (*n* = 8) is shown in a bar diagram. Fluorescence was measured at 1 and 5 hours, respectively, after intramuscular or subcutaneous injection of Alexa Fluor 680–labeled ADDomer (10 μg). Bar color coding is as follows: dark blue, intramuscular at 1 hour; brown, intramuscular at 5 hours; light blue, subcutaneous for 1 hour; red, subcutaneous for 5 hours; black, control (noninjected) mice (*n* = 5). SDs are shown as error bars. Axi, axillary; Bra, brachial; Sci, sciatic; Ing fpr, inguinal; Mes, mesenteric lymph nodes; RLU, relative light unit per pixel.

ADDomer (30 nm) is within the size range (20 to 200 nm) of particles readily drained to lymph nodes, potentiating uptake by antigen-presenting cells and cross-presentation (*5*, *6*). We analyzed ADDomer uptake by human cells, including monocytes and monocyte-derived dendritic cells (MDDCs), confirming efficient internalization (Fig. 2, A and B). We next investigated lymph node distribution in mice injected with fluorescently labeled ADDomer (Fig. 2, C and D). Rapid draining to the right inguinal lymph node was observed irrespective of the mode of administration. No signal was found in the opposite left inguinal node serving as internal control. Mesenteric and axillary nodes evidenced rapid but not lasting signal (Fig. 2D). Our results underscore ADDomer capacity to drain rapidly to the nearest lymph node and to efficiently penetrate human cells, including antigen-presenting lymphocytes.

### Engineering ADDomer to combat emerging infectious diseases

Infectious diseases continue to decimate populations worldwide, and among our means to combat this threat, vaccination is exceptionally powerful. Recent examples that dominated the media are Chikungunya and Zika infectious diseases, transmitted by mosquitoes originally confined to sub-Saharan Africa but increasingly spreading to the Northern Hemisphere. We used our plug-and-play setup to rapidly generate ADDomers displaying multiple copies of immunogenic epitopes from a range of human and livestock viral pathogens, testing the capacity of the insertion loci in the process (fig. S6 and table S2). To assess the flexibility of the ADDomer to harbor large multiepitope insertions, we combined five immunogenic Gumboro-derived epitopes in tandem. Gumboro is caused by infectious bursal disease virus and affects poultry, causing significant economic shortfall, particularly in developing countries. ADDomer evidenced a remarkable plasticity for the insertion of the five tandem Gumboro-derived epitopes in both functionalized VL and RGD loop without compromising integrity (fig. S6, A and B). Insertion in the VL evidenced a blurring of the penton edges, suggesting preferred orientations of the loop structure comprising the Gumboro epitopes. Notably, we successfully inserted a test epitope exceeding 200 amino acid residues in the RGD loop (fig. S6B). Together, our data demonstrate full functionality of the insertion loci we engineered and convey that long sequences containing multiple epitopes can be efficiently displayed at high density on the ADDomer.

**Fig. 3:**
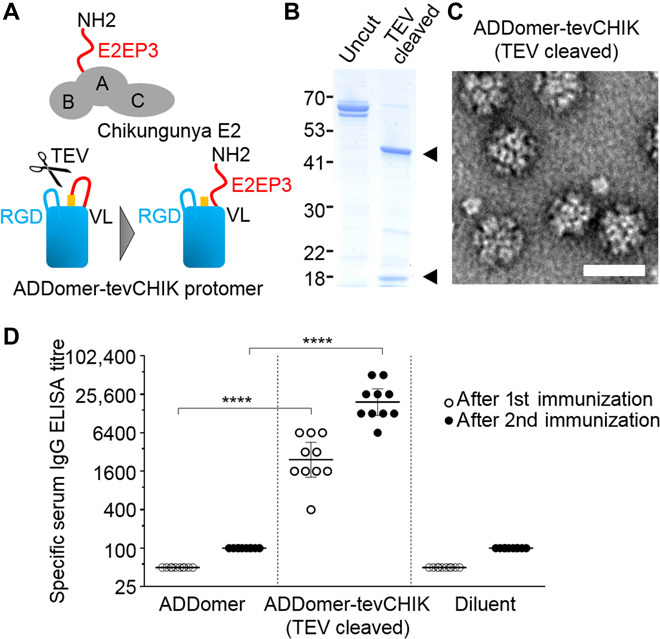
ADDomer immunizations. (**A**) Top: Chikungunya envelope protein E2, composed of domains A, B, and C, is shown in a schematic representation. The major neutralizing epitope E2EP3 (red) comprises the N terminus of E2 with the amino acid sequence NH2-STDKDNFNVYKATRPYLAH. Bottom: This specific configuration of E2E3P was mimicked in ADDomer-tevCHIK as depicted by inserting the E2EP3 sequence (in red), preceded by a TEV protease cleavage site (orange) into the BioBrick cassette in VL of the ADDomer protomer. Cleavage by the protease exposes E2EP3 in a native-like configuration as shown schematically. (**B**) SDS–polyacrylamide gel electrophoresis section showing purified ADDomer-tevCHIK before and after cleavage by TEV protease. Molecular weight marker sizes are marked (left). Appearance of two bands (black triangles) indicates quantitative cleavage. (**C**) Representative negative-stain EM images of TEV-cleaved ADDomer-tevCHIK reveals stable dodecamers. Scale bar, 30 nm. (**D**) This TEV-cleaved sample was used in immunization experiments in mice (*n* = 10 per group). Scatter dot plot diagram depicts the outcome of the immunization experiments, revealing high individual IgG titers specific for Chikungunya E2E3P peptide in enzyme-linked immunosorbent assay (ELISA). Short horizontal line segments are geometric mean titers, and error bars represent the 95% confidence interval. Significant difference (****P < 0.0001) was determined between ADDomer-tevCHICK– and ADDomer-only immunized mice.

Notably, sera of most patients suffering from Chikungunya viral infection react with a specific linear peptide, E2EP3 (*24*). This major neutralizing epitope is located at the very N terminus of the viral E2 glycoprotein, comprising the first 18 amino acids (Fig. 3A and table S2). We inserted E2EP3 into ADDomer to create a VLP vaccine candidate, ADDomer-tevCHIK, with the potential to combat this debilitating disease. To recapitulate the exposed conformation of E2EP3 in Chikungunya virus, we inserted a site for a highly specific protease [tobacco etch virus (TEV) NIa] right in front of the epitope, which, upon cleavage, generates a native-like conformation comprising an exposed N-terminal serine (Fig. 3A). Processing by TEV resulted in quantitative cleavage (Fig. 3B). Negative-stain EM confirmed that, impressively, the ADDomer dodecahedron remained intact notwithstanding multiple (60-fold) polypeptide backbone cuts (Fig. 3C). Immunization experiments compellingly validated our approach. Uncut ADDomer-tevCHIK did not yield E2EP3-specific immunoglobulin titers (fig. S7). In contrast, quantitatively cleaved ADDomer-tevCHIK exposing native-like E2EP3 elicited very strong specific immunoglobulin G (IgG) response concomitant with barely detectable IgM (Fig. 3D and figs. S7 and S8), in excellent agreement with the immune reaction to Chikungunya viral infection. An unrelated nanoparticle scaffold made of polylactic acid harboring the same amount of CHIK peptide epitopes did not result in an immune response above background in mice, suggesting that the ADDomer scaffold itself may have an adjuvanting effect (fig. S7).

## DISCUSSION

In summary, we presented here ADDomer, our plug-and-play multiepitope display platform for antigenic epitopes from different origins, including emerging human viral pathogens or veterinary infectious diseases. We determined ADDomer architecture at near-atomic resolution by cryo-EM, enabled by a heterogeneous computation cluster and created and executed automatically with public cloud infrastructure. Our approach presents a novel, fast, and cost-effective general mechanism to quickly analyze and process cryo-EM data and sets the stage for comprehensive (re)design of the ADDomer particle, also to fine-tune self-adjuvanting potential by modulating sequence plasticity (*25*). ADDomer is easy to modify and produce in high yields using our recombinant system. It is remarkably thermostable, which could render deployment of ADDomer-based vaccines independent of a cold chain, representing a crucial asset. We demonstrate that ADDomer rapidly drains to lymph nodes and efficiently internalizes in immune blood cells. We inserted multiepitope sequences exceeding 200 amino acids without compromising ADDomer integrity, conveying that even folded domains or entire proteins could be displayed. ADDomer can be cleaved multiple times in loops without adverse effects, enabling native-like display of epitopes adopting exposed conformations, compellingly demonstrated here with Chikungunya E2EP3. Notably, modification of ADDomer is not restricted to the engineered loci. The fiber binding cleft could also be used to attach peptides and proteins, tagged with fiber peptide. Moreover, the N-terminal extension was shown to react with specific binder domains (*19*), conferring further capacity. Thus, ADDomer can accommodate hundreds of epitopes, same or different, providing vast opportunity for combinatorial display by a single particle (fig. S9). Last, the central cavity within ADDomer could be used to encapsulate costimulatory molecules. We anticipate that a wide range of applications in biomedical research will benefit from this exceptionally versatile display platform.

## MATERIALS AND METHODS

### ADDomer design

We used the penton base protein from human Ad3 (GenBank Z29487) as a starting point and implemented a BioBrick format (*22*) to functionalize one locus within the VL and two loci in the RGD loop for facile and rapid custom DNA insertion (fig. S1). The insertion loci were flanked by unique restriction sites that were eliminated from elsewhere in the Ad3 penton base encoding gene. To preserve structural integrity of the penton base protomer, the restriction sites were designed in silico to be located outside of and adjacent to known secondary structure elements in the penton base protein. The resulting gene encoding for ADDomer was synthesized, codon-optimized for recombinant expression, and pasted into plasmid pACEBac1 (Geneva Biotech SARL, Switzerland) (*26*) using Bam HI and Hind III restriction sites, giving rise to pACEBac-ADDomer. The primary sequence of the encoded ADDomer is provided in fig. S2. In this study, pACEBac-ADDomer served as the backbone for inserting DNA encoding for selected epitopes into the designed insertion loci in the variable and RGD loops. Primary sequences of the inserted epitopes in this study are listed in table S2.

### Expression and purification

pACEBac-ADDomer and epitope-displaying variants were expressed using the MultiBac baculovirus expression system we developed (fig. S1) (*23*). Briefly, pACEBac-ADDomer and variant plasmids were inserted by Tn7 transposition into an engineered baculoviral genome, EMBacY, in DH10EMBacY cells (*27*). Transfection, baculovirus amplification, storage, and protein expression in Hi5 insect cell suspension cultures (50 ml) were performed following established protocols (*27*), resulting in exceptional yields (fig. S1). The EMBacY baculoviral genome encodes a yellow fluorescent protein (YFP) marker. When the YFP signal reached a plateau, cells were harvested by centrifugation, and pellets were flash-frozen in liquid nitrogen and stored at −20°C. Pellets were resuspended in hypotonic lysis buffer [0.33× phosphate-buffered saline (PBS); 1 ml per 2.5 × 10^7^ cells] supplemented by EDTA-free cOmplete protease inhibitor (Roche, Switzerland) and lysed by three cycles of freezing and thawing. Lysate was cleared from debris by centrifugation (6000*g* for 30 min), and the cleared supernatant was subjected to sucrose gradient (15 to 40%) centrifugation (*15*). Fractions containing ADDomer (30 to 40% sucrose) were pooled and dialyzed against buffer A [10 mM Hepes (pH 7.4) and 50 mM NaCl]. ADDomer was further purified by ion exchange chromatography using a High Q column (Bio-Rad) using buffer A and applying a linear salt gradient (50 to 500 mM NaCl). Fractions containing highly purified ADDomer (330 to 400 mM NaCl) were pooled and stored at ambient temperature or frozen (−80°C). For animal studies, ADDomer particles were further purified using Detoxi-Gel (Thermo Fisher Scientific) to remove endotoxins, followed by dialysis against PBS.

Following purification, absorbance spectroscopy measurement showed an OD_260nm_ (optical density at 260 nm)/OD_280nm_ ratio below 0.6, evidencing lack of nucleic acid contamination. Before injection into mice, endotoxin levels were quantified using Endosafe PTS (Charles River) evidencing 754 EU (endotoxin units)/mg for ADDomer-tevCHIK and 247 EU/mg for ADDomer, respectively. In our injections, we thus were below 30 EU, the common cutoff value for testing adjuvant properties of any recombinant antigen in a mouse model.

### Thermostability measurements

Highly purified ADDomer sample was stored in PBS at room temperature. Thermal shift experiments were performed at an ADDomer concentration of 3 mg/ml in PBS, using a ThermoFluor assay (*28*). ADDomer sample was diluted to 1 mg/ml and aliquoted. Identical sample aliquots were stored for several weeks at room temperature, frozen at −20°C overnight followed by thawing, and incubated at 45°C for 1 hour, respectively. Sample integrity was assessed at each intervention by negative-stain EM (fig. S5).

### Negative-stain EM

Negative-stain EM was performed with purified ADDomer (0.1 to 0.5 mg/ml) dialyzed into 25 mM Hepes (pH 7.5), 150 mM NaCl, and 2 mM EDTA. Sample was stained with 3% uranyl acetate. Micrographs were recorded using a 200-kV FEI Tecnai T20 microscope with an Eagle 4k × 4k charge-coupled device camera.

### Cryo-EM and data collection

Five microliters of purified ADDomer (0.1 mg/ml) was applied to glow-discharged holey carbon grids (Quantifoil, R 2/2 300 mesh). Samples were incubated on the grid for 2 s and blotted for 0.4 s at 90% relative humidity and 18°C inside a Leica EM GP, before plunge-freezing in liquid ethane. Cryo-EM data were collected at 200 kV with a FEI Talos Arctica microscope equipped with a Gatan K2 direct electron detector and an energy filter using automated acquisition software (EPU). A total of 1060 dose-fractionated movies each containing 32 frames (0.25 s per frame) with an accumulated total dose of 42 *e*^−^/Å^2^ were recorded at a nominal magnification of ×130,000 corresponding to a pixel size of 1.06 Å. Images were recorded with a defocus range of −0.8 to −3.2 μm (table S1).

### Image processing

Image processing was performed with the RELION 2.1 software package (*29*). The micrographs were motion-corrected using MotionCorr2 (*30*), and contrast transfer function (CTF) information was determined using ctffind4.1 (*31*). Together, 262 micrographs with CTF rings extending beyond 4.6 Å were selected for further processing. A total of 3600 particles were boxed using RELION autopicking software. After two-dimensional (2D) classification (fig. S3C) and 3D classification with imposed icosahedral symmetry, initial 3D autorefinement led to a reconstruction of ~4.2-Å resolution. Further rounds of 3D classification/refinement were carried out on polished particles before using postprocessing for masking and automatic B factor sharpening. The resolution of the final map was determined to be 3.5 Å based on the Fourier shell correlation (FSC) 0.143 cutoff criterion (fig. S3D) (*32*). Local resolution was calculated using local resolution estimation program in RELION (fig. S3E).

Processing and 3D classification of ADDomer were performed using public cloud resources provided by the Oracle Cloud Infrastructure. Using this rather than an institutional cluster allowed a faster turnaround time of analysis. Because of the particular nature of the computational work carried out by the steps within RELION, a heterogeneous cluster was prepared with both central processing unit (CPU)– and graphical processing unit (GPU)–focused resources. A pipeline of workloads was created, allowing the work best suited to each compute type to use just the resources needed and to automatically release compute resources when no longer needed. This permitted parts of the workflow to use lower-cost CPU resources and to burst to GPU only for those parts that would benefit from GPU optimization, reducing overall cost while increasing total data throughput. The total cost for running the image processing pipeline on the cloud was approximately £200.

### Model building and refinement

Homology modeling was performed using I-TASSER (*33*) using the human adenovirus Ad3 structure as template (*17*). The model was adjusted to fit into the map manually using COOT (*34*) before further iterative positional and B factor refinement in real space using Phenix Real-Space refinement software (*35*). Final adjustments were carried out in COOT before evaluating the model using MolProbity (*36*). Refinement statistics are summarized in table S1.

### Cell culture and differentiation

HeLa cells were cultured in Dulbecco’s modified Eagle’s medium supplemented with 10% fetal calf serum (FCS) (Thermo Fisher Scientific) at 37°C under 5% CO_2_. Monocytes were purified by centrifugation on Ficoll (Sigma-Aldrich) for 20 min at 800*g*, and mononucleated cells were subsequently passed through EasySep magnetic beads (STEMCELL Technology). Monocyte purity was verified by flow-cytometry by means of CD14 labeling. MDDCs were generated by cultivating monocytes in RPMI 1640 FCS medium supplemented with granulocyte-macrophage colony-stimulating factor at 100 ng/ml and interleukin-4 at 25 ng/ml for 6 days. Differentiation to MDDCs was verified by FACS based on CD14/CD209 labeling.

### Imaging

HeLa cells were grown on a glass coverslip and incubated for 1 hour at 37°C with unlabeled ADDomer at 20 μg/ml. Subsequent to methanol fixation, rabbit serum raised against human Ad3 penton base protein, diluted 1:1000 in PBS, was applied, followed by Cy5-labeled goat anti-rabbit antibody (Jackson ImmunoResearch), diluted 1:500 in PBS. Nuclei were stained by the DNA dye 4′,6-diamidino-2-phenylindole.

Monocytes and MDDCs from healthy anonymous donors were obtained from the National Blood Service [Etablissement Francais du Sang (EFS), La Tronche] after written consent following approval by the EFS. ADDomer (1 mg/ml) was labeled with Alexa Flour 488 dye (Invitrogen) following the manufacturer’s recommendations. Unbound dye was removed by extensive dialysis against PBS overnight. About 10^5^ monocytes or MDDCs, respectively, were incubated for 1 hour at 37°C with 10 μg of labeled ADDomer. Cells were applied to glass coverslips and analyzed using differential interference contrast and green fluorescence channels in an Olympus IX81 inverted microscope.

### Biodistribution studies

ADDomer was labeled with Alexa Fluor 680 dye (Invitrogen) following the same procedure as above for Alexa Flour 488 labeling. Alexa Flour 680–labeled ADDomer sample was injected in 9-week-old female BALB/cJRj mice (Janvier Labs, France). Four control mice (no injection) were used for tissue autofluorescence quantifications. Mice were anesthetized (isoflurane/air, 4% for induction and 1.5% thereafter), and injections were performed either subcutaneously (*n* = 8; right flank; 100 μl; 0.1 mg/ml) or intramuscularly (*n* = 8; right quadriceps; 12 μl; 0.85 mg/ml). Mice were euthanized at either 1 hour (*n* = 4 per group) or 5 hours (*n* = 4 per group) after injection. Main lymph nodes (mesenteric, sciatic, axillary, inguinal, and brachial) were sampled and imaged using FLUOBEAM700 (Fluoptics, France) with 680-nm excitation and LP (Long Pass) 700-nm emission. Fluorescence radiance from each lymph node was quantified in relative light units per pixel per 100 ms.

### Immune studies

#### Initial test immunizations

Six-week-old female BALB/c mice were subcutaneously injected, and sera were collected and treated as described previously (*37*). Immunizations were carried out with 10 μg each of ADDomer, ADDomer-tevCHIK (uncut), or ADDomer-tevCHIK quantitively cleaved by TEV protease, respectively. Specific antibodies in sera were detected by enzyme-linked immunosorbent assay (ELISA) in duplicate using 96-well plates coated with CHIK peptide (0.1 μg/ml). Horseradish peroxidase (HRP)–conjugated goat anti-mouse IgG 1030-05 (SouthernBiotech) was used for detection [concentration of 0.1 μg/ml in PBS with 1% bovine serum albumin (BSA)] for 1 hour at 37°C, supplemented with 100 μl of tetramethylbenzidine (TMB) substrate (Becton Dickinson) per well. Reactions were stopped by adding H_2_SO_4_ (0.2 N), and OD was measured in a microplate reader (Thermo Fisher Scientific) at 450 nm (fig. S7).

#### ADDomer-tevCHIK immunization experiments

Ten B6D2F1 mice per group received twice (study days 1 and 15) subcutaneously 40 μg per mouse of ADDomer (group 1) or quantitatively cleaved ADDomer-tevCHIK (group 2). Control mice were immunized twice with the buffer diluent. Mice were observed cage-side for clinical symptoms. Mice immunized with ADDomer (group 1) and ADDomer-tevCHIK (group 2) developed high serum IgG titers (<12,800) after the first and second immunization. Mice immunized with diluent did not induce ADDomer-specific IgG. IgM and IgG titers were <100 in serum samples collected before immunization. All mice immunized with ADDomer-tevCHIK sample developed CHIK-specific IgG after the first immunization (GMT, 2425; titer range, 400 to 6400). After the second immunization, IgG titers substantially increased (GMT, 19,400; titer range, 6400 to 51,200). Mice immunized with ADDomer (empty) and diluent did not develop CHIK-specific IgG titers. In contrast, only 1 of 10 mice immunized with ADDomer-tevCHIK developed detectable CHIK epitope–specific IgM after the first (titer, 1:50) and second (titer, 1:100) immunization. No CHIK-specific IgM could be determined in mice immunized with ADDomer or diluent.

Blood was collected by the facial vein technique at days 1, 14, and 35. Briefly, the mouse was gently and securely restrained in the operator’s nondominant hand. The hairless freckle on the side of the jaw was located and pricked with a sharp lancet held by operator’s free hand (sharp end of the lancet points at the far side of the mouse’s face, at the base of the far ear, or at the base of the far side of the mouth). About four to seven drops of blood were collected in an Eppendorf tube, and the mouse was released into its cage. Blood samples were centrifuged twice in a bench centrifuge for 5 min at 10,000*g* at 4°C, and collected serum was stored below −18°C before analyses. An ELISA protocol was developed using streptavidin-coated plates and the biotinylated CHIK peptide (table S2) following established protocols (Thermo Fisher Scientific). Briefly, streptavidin-coated plates were washed three times with 200 μl of wash buffer [25 mM tris-HCl (pH 7.2), 150 mM NaCl, 0.1% BSA, and 0.05% Tween 20]. Next, 100 μl of biotinylated CHIK peptide dissolved in dimethyl sulfoxide and diluted in PBS to a final concentration of 2.5 μg/ml in PBS was added to each well. Plates were incubated for 2 hours on a plate shaker at room temperature. After extensive washing, twofold serially diluted serum samples were added (100 μl per well). Plates were incubated for 1 hour with occasional shaking at room temperature. After three additional washing steps, 100 μl of goat anti-mouse IgM or HRP-conjugated IgG antibody (diluted 1:2000; Sigma-Aldrich), respectively, was added to each well, and plates were incubated for 45 min at room temperature. Plates were again extensively washed before adding 100 μl of color substrate (TMB) per well, followed by shaking. The color reaction was stopped with H_2_SO_4_ (0.2 N), and OD was measured at 450 nm. To determine serum IgM or IgG titers, a cutoff value was defined as mean absorption value of negative blank samples plus three times the SD.

### In vivo experimentation statement

In vivo experiments were carried out in France at the Institute for Advanced Biosciences (Grenoble) and the Institute of Biology and Chemistry of Proteins (Lyon). These laboratories are certified by the French Ministry of Research and Education Superior and regularly monitored by the French Departmental Directorate for the Protection of Population (approval numbers C 69 123 0303 and C38 516 10001). Immunization studies were carried out at the Institute of Virology, Biomedical Research Centre, Slovak Academy of Sciences (Bratislava). Animal care was in compliance with the standard operation procedures of the Institute of Virology and the European Convention for the Protection of Vertebrate Animals used for experimental and other scientific purposes (ETS 123) with approval from the Veterinary State Administration, Slovak Republic (Statna veterinarna a potravinova sprava Slovenskej republiky), and institutional ethical committee. All animal experiments were carried out according to the rules in force and in the respect of the ethics of the animal experimentation.

### Statistics

Statistical significance was determined by calculating SDs following standard mathematical formulae (unpaired *t* test with Welch’s correction of log-transformed titer values).

## Supplementary Material

http://advances.sciencemag.org/cgi/content/full/5/9/eaaw2853/DC1

Download PDF

Synthetic self-asembling ADDomer platform for highly efficient vaccination by genetically encoded multiepitope display

## SUPPLEMENTARY MATERIALS

Supplementary material for this article is available at http://advances.sciencemag.org/cgi/content/full/5/9/eaaw2853/DC1 Fig. S1. ADDomer BioBrick design and expression. Fig. S2. ADDomer primary sequence. Fig. S3. EM of ADDomer. Fig. S4. Quality of the ADDomer map and model. Fig. S5. ADDomer thermotolerance. Fig. S6. ADDomer: Genetically encoded multiepitope display. Fig. S7. Initial immunization experiments. Fig. S8. Individual specific IgM serum titers. Fig. S9. ADDomer functionalization. Table S1. Cryo-EM data collection, refinement, and validation statistics. Table S2. ADDomer epitope sequences. References (*38*–*43*)
