# Supplementary material for: Synthetic self-assembling ADDomer platform for highly efficient vaccination by genetically encoded multiepitope display
Source: Sci Adv. 2019 Sep 25;5(9):eaaw2853. doi: 10.1126/sciadv.aaw2853 (PMC6763337; doi:10.1126/sciadv.aaw2853)
Supplement: http://advances.sciencemag.org/cgi/content/full/5/9/eaaw2853/DC1 [file supp_5_9_eaaw2853__index.html]

Science Advances | Science AdvancesAAASSearchScience AdvancesMenu

## Supplementary Materials

**This PDF file includes:**

- Fig. S1. ADDomer BioBrick design and expression.
- Fig. S2. ADDomer primary sequence.
- Fig. S3. EM of ADDomer.
- Fig. S4. Quality of the ADDomer map and model.
- Fig. S5. ADDomer thermotolerance.
- Fig. S6. ADDomer: Genetically encoded multiepitope display.
- Fig. S7. Initial immunization experiments.
- Fig. S8. Individual specific IgM serum titers.
- Fig. S9. ADDomer functionalization.
- Table S1. Cryo-EM data collection, refinement, and validation statistics.
- Table S2. ADDomer epitope sequences.
- References (*38*–*43*)

Download PDF

**Files in this Data Supplement:**

- Adobe PDF - aaw2853\_SM.pdf
