# Supplementary material for: Synthetic self-assembling ADDomer platform for highly efficient vaccination by genetically encoded multiepitope display
Source: Sci Adv. 2019 Sep 25;5(9):eaaw2853. doi: 10.1126/sciadv.aaw2853 (PMC6763337; doi:10.1126/sciadv.aaw2853)
Supplement: Download PDF [file aaw2853_SM.pdf]

Science Advances
AAAS

advances.sciencemag.org/cgi/content/full/5/9/eaaw2853/DC1

Supplementary Materials for

Synthetic self-assembling ADDomer platform for highly efficient vaccination by genetically encoded multiepitope display

Charles Vragniau, Joshua C. Bufton, Frédéric Garzoni, Emilie Stermann, Fruzsina Rabi, Céline Terrat, Mélanie Guidetti, Véronique Josserand, Matt Williams, Christopher J. Woods, Gerardo Viedma, Phil Bates, Bernard Verrier, Laurence Chaperot, Christiane Schaffitzel*, Imre Berger*, Pascal Fender*

*Corresponding author. Email: pascal.fender@ibs.fr (P.F.); imre.berger@bristol.ac.uk (I.B.); cb14941@bristol.ac.uk (C.S.)

Published 25 September 2019, *Sci. Adv.* **5**, eaaw2853 (2019)
DOI: 10.1126/sciadv.aaw2853

This PDF file includes:

Fig. S1. ADDomer BioBrick design and expression.
Fig. S2. ADDomer primary sequence.
Fig. S3. EM of ADDomer.
Fig. S4. Quality of the ADDomer map and model.
Fig. S5. ADDomer thermotolerance.
Fig. S6. ADDomer: Genetically encoded multiepitope display.
Fig. S7. Initial immunization experiments.
Fig. S8. Individual specific IgM serum titers.
Fig. S9. ADDomer functionalization.
Table S1. Cryo-EM data collection, refinement, and validation statistics.
Table S2. ADDomer epitope sequences.
References (*38–43*)

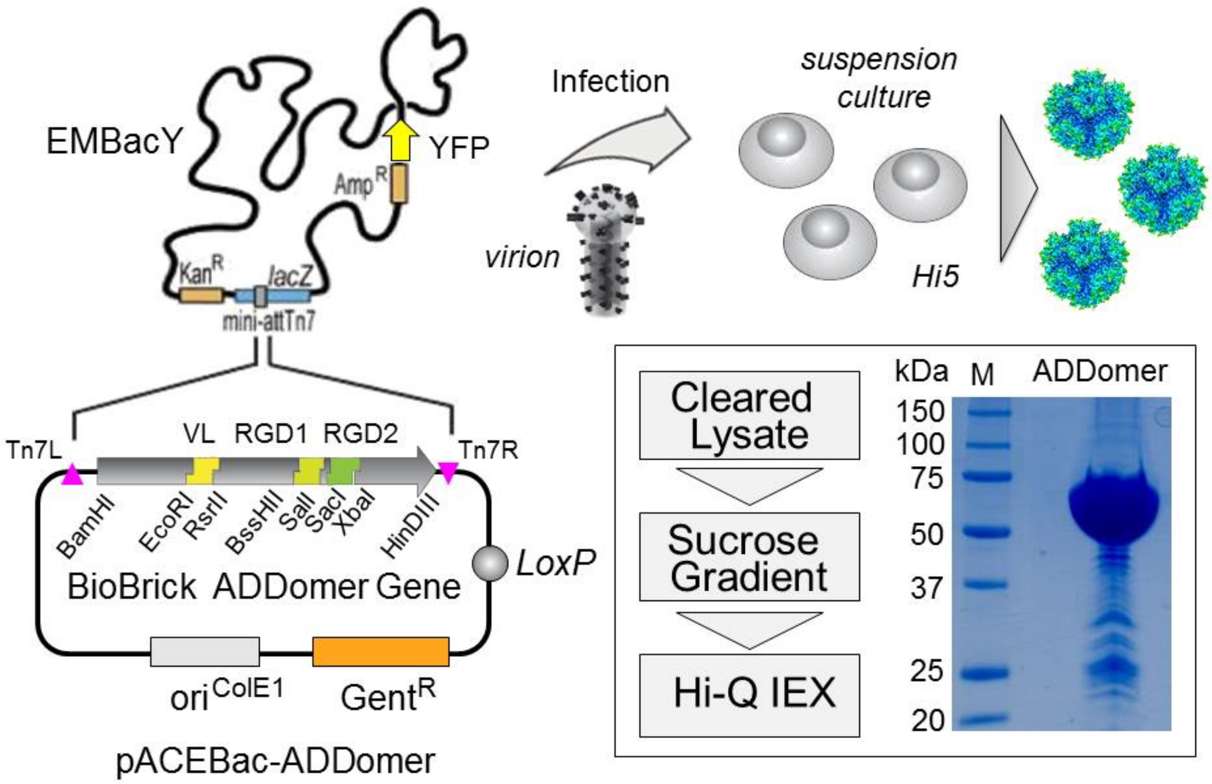

Fig. S1. ADDomer BioBrick design and expression. A synthetic gene encoding ADDomer was designed in BioBrick format[22] with functionalized insertion *loci*, one in the original variable loop and two in the original RGD loop (left, bottom). BioBrick parts are DNA sequences which conform to a restriction-enzyme assembly standard originally developed for engineering synthetic DNA circuits from defined DNA blocks using a collection of unique restriction enzymes, thus maintaining spacing between individual DNA elements and preserving reading frames[22]. This synthetic DNA was pasted in pACEBac1 plasmids[26] to yield pACEBac-ADDomer and inserted by Tn7 transposition into the EMBacY baculoviral genome[26] (top, left). Live virions were generated following established protocols[27] and ADDomer produced in Hi5 insect cells in suspension culture. The ADDomer purification process (bottom, right) comprises of a sucrose gradient centrifugation step of the clarified lysate followed by ion exchange chromatography (IEX) using a Hi-Q column. A Coomassie-stained SDS-PAGE section of purified ADDomer is shown (inset). VL, insertion site engineered in variable loop; RGD1 and RDG2, insertion sites in RGD loop; Tn7L,Tn7R and mini-attTn7, recognition and attachment sequences for Tn7 transposase; LoxP, site-specific recombination site for Cre enzyme[26,27]; Gent^R, Gentamicin resistance gene; Kan, Kanamycin; Amp, Ampicillin; LacZ, gene for blue/white screening of composite genome; ori^{ColE1}, replication origin, YFP, yellow fluorescent protein; M, molecular weight marker (sizes given in kDa). All ADDomer variants were produced and purified using this protocol.

```
        10         20         30         40         50         60
MRRRAVLGGA VVYPEGPPPS YESVMQQQAA MIQPPLEAPF VPPRYLAPTE GRNSIRYSEL

        70         80         90        100        110        120
SPLYDTTKLY LVDNKSADIA SLNYQNDHSN FLTTVVQNND FTPTEASTQT INFDERSRWG

       130        140        150        160        170        180
GQLKTIMHTN MPNVNEYMFS NKFKARVMVS RKAPEGEFVT VNDGPVNDTY DHKEDILKYE

       190        200        210        220        230        240
WFEFILPEGN FSATMTIDLM NNAIIDNYLE IGRQNGVLES DIGVKFDTRN FRLGWDPETK

       250        260        270        280        290        300
LIMPGVYTYE AFHPDIVLLP GCGVDFTESR LSNLLGIRKR HPFQEGFKIM YEDLEGGNIP

       310        320        330        340        350        360
ALLDVTAYEE SKKDTTTARE TTTLAVAEET SEDVDDDITR GDTYITELEK QKREAAAAEV

       370        380        390        400        410        420
SRKKELKIQP LEKDSKSRSY NVLEDKINTA YRSWYLSYNY GNPEKGIRSW TLLTTSDVTC

       430        440        450        460        470        480
GAEQVYWSLP DMMQDPVTFR STRQVNNYPV VGAELMPVFS KSFYNEQAVY SQQLRQATSL

       490        500        510        520        530        540
THVFNRFPEN QILIRPPAPT ITTVSENVPA LTDHGTLPLR SSIRGVQRVT VTDARRRTCP

       550
YVYKALGIVA PRVLSSRTF
```

Fig. S2. ADDomer primary sequence. Functionalized insertion sites in variable loop (VL) and RGD loop (RGD1, RGD2) are highlighted by color coding as in fig. S1A. Amino acid residues encoded by restriction enzymatic sites corresponding to BioBrick design are boxed in red. In between insertion sites, native adenovirus sequences were maintained, notably the RGD tripeptide sequence (orange) to preserve integrin mediated internalization of ADDomer.

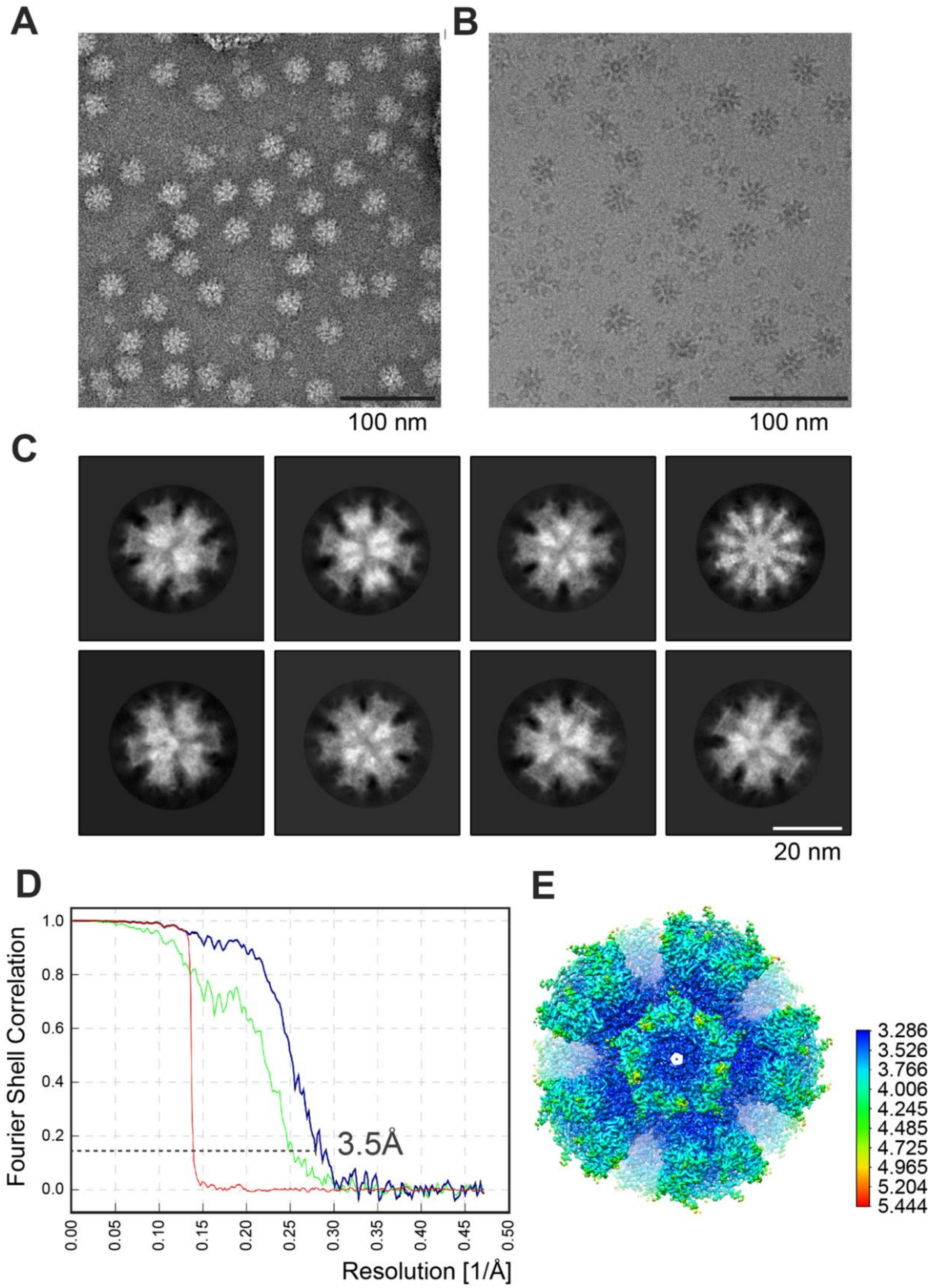

Fig. S3. EM of ADDomer. **(a)** Negative stain micrograph showing purified ADDomer. **(b)** A representative cryo-EM micrograph with ADDomer particles. During grid freezing some dissociation of ADDomer particles into pentons is observed. **(c)** Reference-free 2D class averages from RELION 2.1 [29]. **(d)** The Fourier Shell correlation (FSC) curve after gold-standard refinement of 1,758 particles (blue curve). The FSC = 0.143 criterion[32] indicates an overall resolution of 3.5 Å. Green curve: FSC curve of unmasked maps; red curve: FSC curve of phase randomized masked maps. **(e)** Local resolution of the final ADDomer cryo-EM map calculated in RELION 2.1. The core of the complex is resolved at 3.2 Å whereas peripheral parts comprising the VL and RGD loops have a lower resolution of ~3.7-4.8 Å.

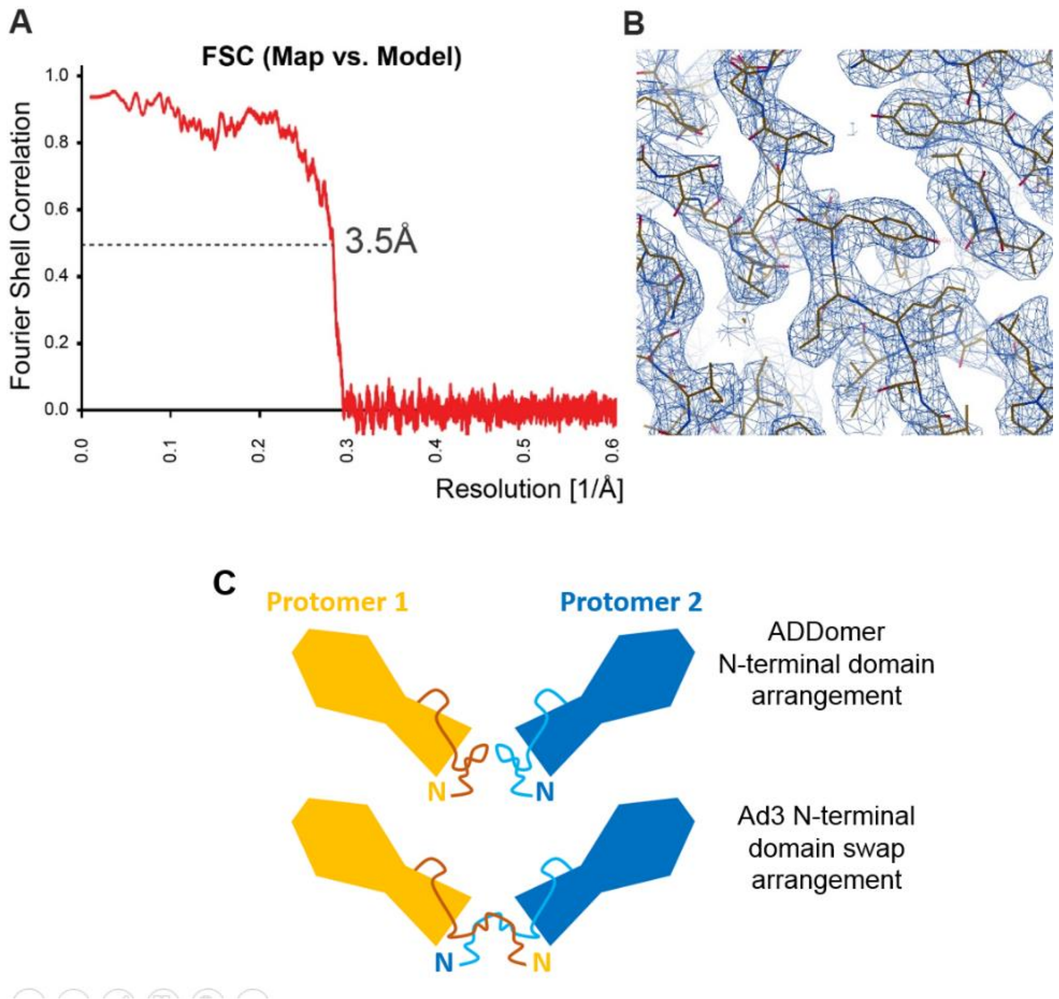

Fig. S4. Quality of the ADDomer map and model. (a) FSC curve calculated between the atomic model and the final EM map. The map/model FSC at 0.5 reaches a resolution of 3.5 Å. The FSC between the two independent half maps from cryo-EM reached the same resolution at 0.143 (fig. S1D), indicating a very good overall quality of fitting. **(b)** Representative EM density of the ADDomer containing the refined atomic model, highlighting the excellent quality of the experimental map. (c) Schematic drawing comparing N-terminal strand arrangement of the penton base protomers in our ADDomer cryo-EM structure (top) and the Ad3 crystal structure (bottom). Protomers from two distinct pentons are colored orange and blue, respectively. N denote N-termini. The N-terminal domains in the Ad3 crystal structure are swapped between the protomers. No swap is observed in the ADDomer.

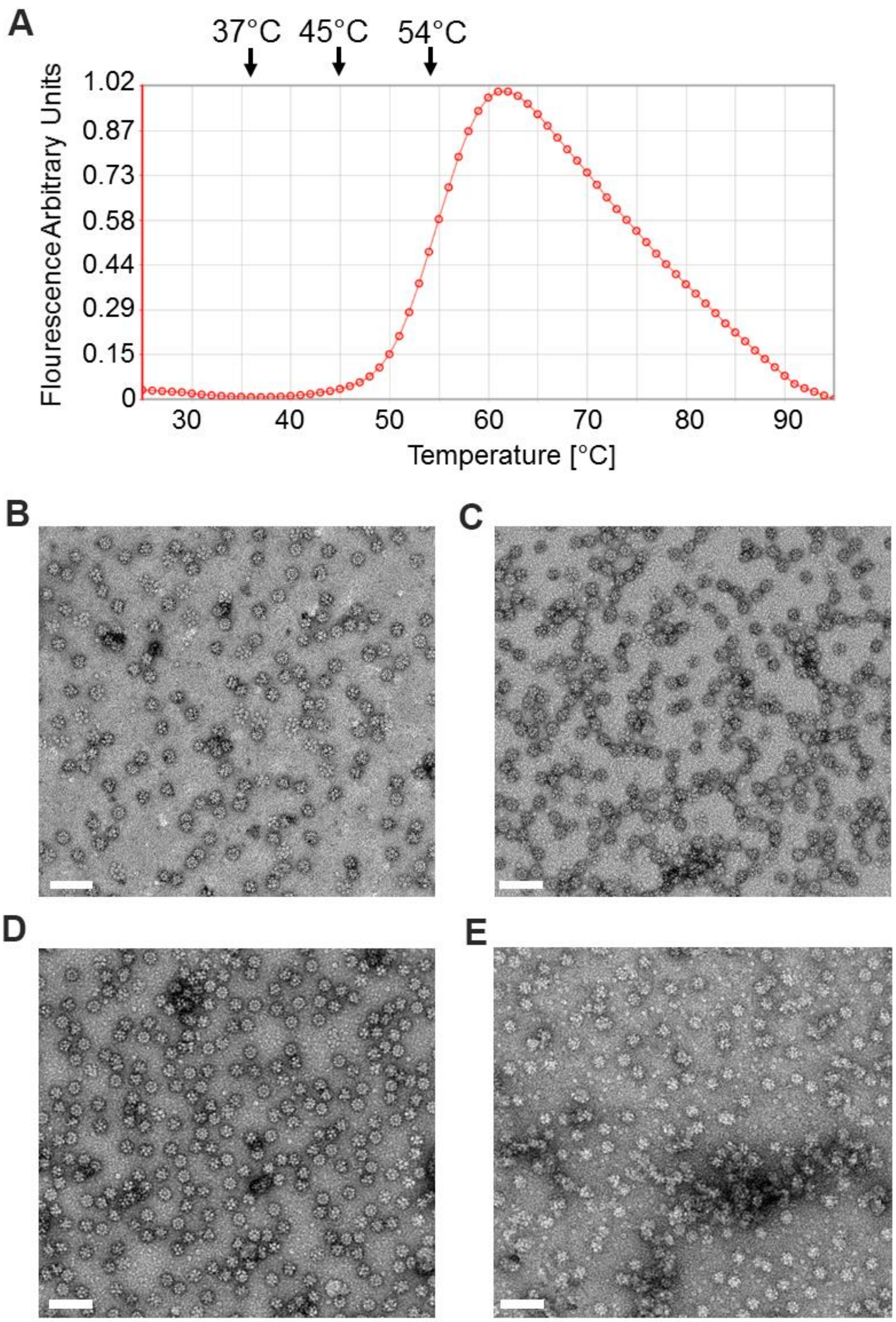

Fig. S5. ADDomer thermotolerance. (**a**) Thermal stability of ADDomer was assessed by thermal unfolding measurements[28] exhibiting exceptional thermotolerance (melting temperature Tm=54^{0}C). (**b-e**) ADDomer integrity was assessed by negative-stain EM. Freshly purified ADDomer is shown in (**b**); ADDomer kept at room temperature (RT) for a month in (**c**); ADDomer kept at RT for a month, frozen and thawed in (**d**); ADDomer kept at RT for a month, frozen and thawed and then incubated at 45^{0}C for 1 hour in (**e**). Scale bars (100nm) are provided.

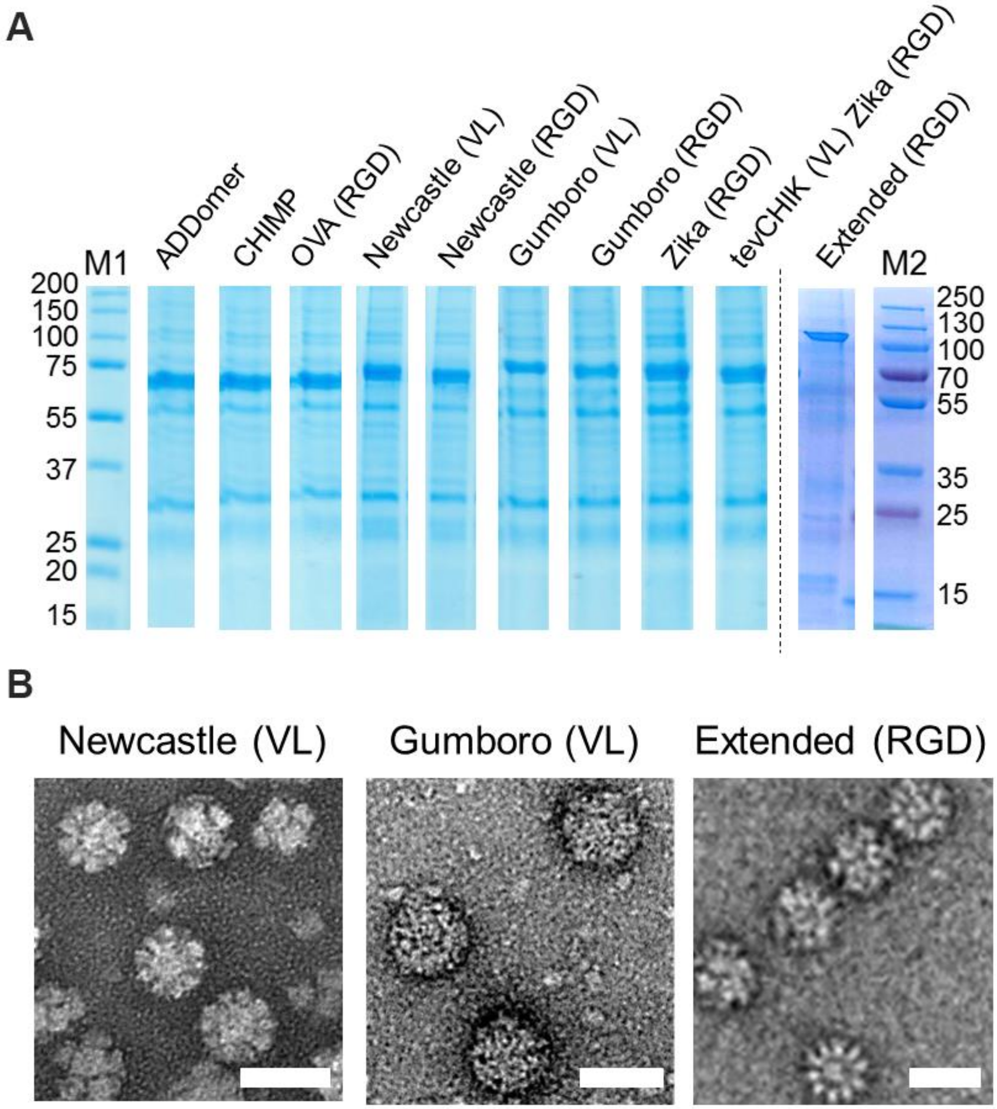

Fig. S6. ADDomer: Genetically encoded multiepitope display. (**a**) Coommassie-stained SDS-PAGE sections of ADDomer and ADDomer variants produced in this study are shown. VL and RGD specify epitope insertion into functionalized sites in variable loop or RGD loop, respectively (Supplementary Table 2). CHIMP, chimpanzee; OVA, melanoma model epitope; CHICK, Chikungunya major neutralizing epitope; tev, tobbaco etch virus NIa proteolytic site; M1 and M2, molecular weight markers (sizes given in kDa). (**b**) Integrity of ADDomer variants were assessed by negative-stain electron microscopy. Scale bars (30 nm) are provided.

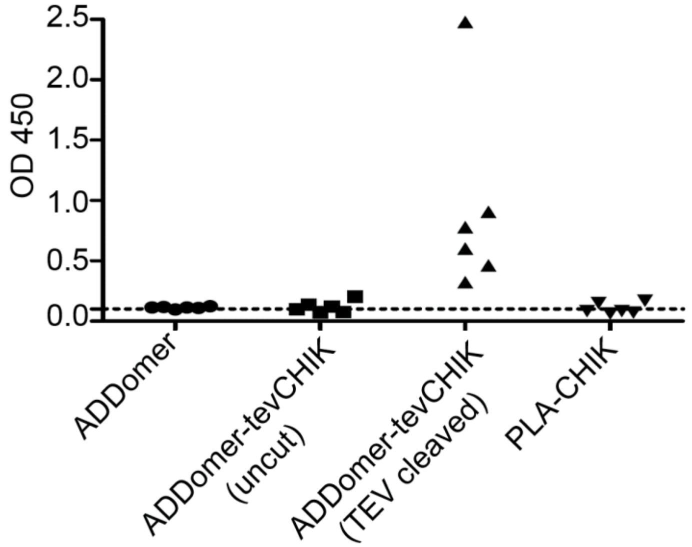

Fig. S7. Initial immunization experiments. Mice were subcutaneously injected with ADDomer (far left), unprocessed ADDomer-tevCHIK (middle, left), ADDomer-tevCHIK quantitatively processed with TEV protease (middle, right) as well as with an organic polymer CHICK peptide conjugate, PLA-CHICK (far right). Processed ADDomer-tevCHIK exposes the Chikungunya neutralizing epitope in a nature-like unconstrained conformation. PLA-CHICK peptide conjugate was prepared using a protocol for conjugation described previously[38,39]. OD 450, optical density measured at wavelength 450 nm.

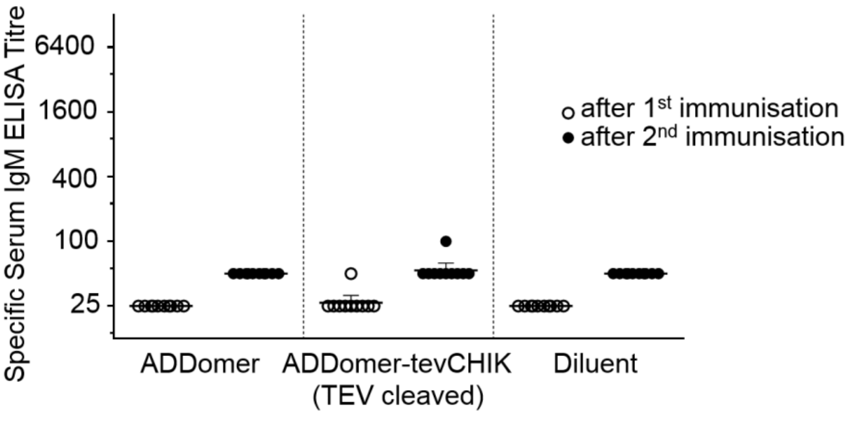

Fig. S8. Individual specific IgM serum titers. Mice were immunised twice with 40µg/mouse of ADDomer or ADDomer-tevCHIK. Control mice were immunised with diluent. Shown are individual serum IgM titres after two subcutaneous immunizations of B6D2F1 hybrid mice. Short horizontal line segments are geometric mean titres and error bars represent the 95% confidence interval. OD 450, optical density at 450 nm wavelength. The baseline difference is caused by different starting sample dilutions after 1st and 2nd immunisation.

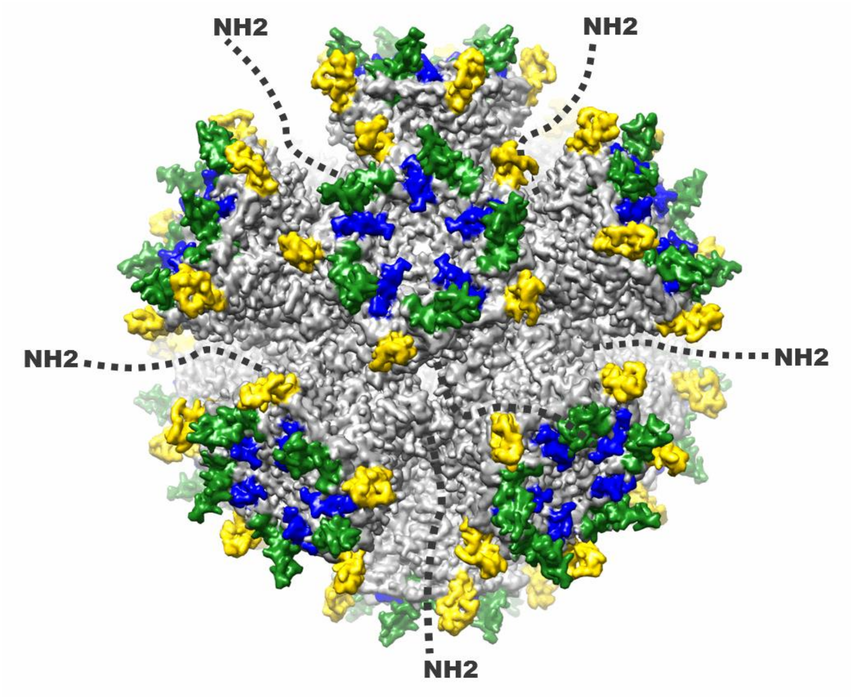

Fig. S9. ADDomer functionalization. Multiple *loci* for displaying epitopes are shown. VL loop insertion sites are colored yellow, RGD loop insertion sites green, fiber binding cleft blue. In addition, the unstructured N-termini of the protomers (dark gray, dashed) extending out through gaps in between the pentons, can have further epitopes attached by specific binding domains. Every locus can accommodate several epitopes, same or different, resulting in vast possibilities for combinatorial display of hundreds of epitopes at high density on a single ADDomer particle.

Table S1. Cryo-EM data collection, refinement, and validation statistics.

**Data collection and processing**	
Voltage (kv)	200
Pixel size (Å)	1.06
Electron exposure ($e^{-}/Å^2$)	42
Defocus range (μm)	-0.8 to -3.2
Symmetry imposed	Icosahedral I4
Initial particle images	3,600
Final particle images	1,758
Resolution (Å, FSC = 0.143 criterion)	3.5
Map-sharpening B factor ($Å^2$)	-81.1317
**Atomic Model**	
Template for homology modelling	4AR2
**Model composition**	
Non-hydrogen atoms	0
Protein residues	26460
**RMS deviations**	
Bond lengths (Å)	0.0075
Angles	1.35°
**Validation**	
MolProbity score	1.73
Clashscore	2.90
Poor rotamers (%)	0.00
C-beta deviations	0
**Ramachandran plot**	
Favoured (%)	85.26
Allowed (%)	14.74
Outlier (%)	0.00

Table S2. ADDomer epitope sequences.

Epitope*	Primary sequence	Context
Chikungunya[†]	STKDNFNVYKATRPYLAH [24]	Human infectious disease epitopes
Zika	DAHAKRQTVVVLGSQEGAV [40]	
OVA	SIINFEKL [19]	Human melanoma model epitope
Newcastle	PDEQDYQIRMAKS [41]	Livestock infectious disease epitopes
Gumboro	MPKTHNSGRSNVDGGGSTLHLPHLWRPLSGGGSHNAKYVSAESWGGGSHPDSIHPFLASPGGGSDTLHGHGFTNWF [42]	
Extended	ENLFYQSEQGGGGAGGGNNSGSGAEENSNAAAAAMQPVEDMNDHAIRGDTFATRAEEKRAEAEAAAEAAAPAAQPEVEKPQGGSGGSGGAKIEAATAAAEAKANIVASDSTRVANAGEVRGDNFAPTPVPTAESLLADVSEGTDGGSGGSGGTETTTLAVAEETSEDDDITRGDTYITEKQKREAAAAEVKK [43]	Test epitope (fused RGD loop sequences from human Adenovirus serotypes Ad2, Ad11 and Ad3, separated by flexible linker sequences)

** All inserted amino acid sequences comprise the epitope flanked by linker residues GGSG (N-term) and GSGG (C-term) for added flexibility.*

† Chikungunya epitope comprises in addition to N-terminal linker residues GGSG a TEV protease cleavage site directly preceding the epitope (GGSGENLYFQ'S..., TEV recognition site underlined, S corresponds to first serine in epitope sequence).
